# Brentuximab-Induced Peripheral Neuropathy in the Setting of Radiation-Induced Brachial Plexopathy

**DOI:** 10.7759/cureus.103554

**Published:** 2026-02-13

**Authors:** Auden Gu, Yehuda Masturov, Mark Rekant, Andrew Miller

**Affiliations:** 1 Sidney Kimmel Medical College, Thomas Jefferson University, Philadelphia, USA; 2 Research, Philadelphia Hand to Shoulder Center, Philadelphia, USA; 3 Orthopedic Surgery, Philadelphia Hand to Shoulder Center, Philadelphia, USA

**Keywords:** brachial plexus neuropathies, brentuximab vedotin, cancer rehabilitation, case report, hodgkin’s lymphoma, peripheral neuropathy, radiation-induced brachial plexopathy

## Abstract

Peripheral neuropathy is characterized by progressive, debilitating sensory loss and motor dysfunction. Radiation-induced brachial plexopathy (RIBP) causes significant motor disability in approximately 1.2% of patients receiving radiotherapy. The incidence of chemotherapy-induced peripheral neuropathy (CIPN) has increased with the expanded use of chemotherapeutic agents. Brentuximab vedotin (BV), an anti-CD30 monoclonal antibody approved for Hodgkin’s lymphoma (HL) in the pediatric population, induces sensory neuropathy. The double crush phenomenon describes two sites of nerve compression along a peripheral nerve, resulting in greater neurological dysfunction than at one site. One example is when CIPN is concomitant with RIBP, although these cases are not well described in the literature.

A patient with BV-induced CIPN with concomitant RIBP was seen at our tertiary referral center, which specializes in complex upper extremity care. This case contributes to the literature by describing the diagnosis and clinical characteristics of chemoradiotherapy-related neuropathies and their subsequent management.

A 16-year-old male with a history of refractory HL presented with significant right upper extremity weakness. Nine months prior, he had completed 14 rounds of stereotactic body radiotherapy; seven months prior, he had begun maintenance therapy with BV. Within four months of his visit, weakness progressed to his bilateral lower extremities and contralateral upper extremity with mild distal sensory loss. Electromyography and nerve conduction studies evaluating peripheral nerve function demonstrated an asymmetric, non-length-dependent, predominantly motor-sensory polyradiculoneuropathy. MRI of the brachial plexus and cervical spine demonstrated asymmetric thickening of the right C5-C8 nerve roots and trunks with a high T2 signal and subtle enhancement with contrast and no infiltrative neoplastic lesion. Treatment with oral prednisone for four weeks resulted in objective clinical improvement three months after treatment was initiated.

Neurotoxic chemotherapeutic agents may potentiate the effects of radiotherapy, resulting in progressive sensory symptoms. BV neurotoxicity may be related to autoimmunity or inhibition of axonal transport. The double crush phenomenon accounts for atypical presentations in patients receiving local radiotherapy or chemotherapeutic agents. Further studies are required to characterize the double crush phenomenon in cancer patients.

## Introduction

Peripheral neuropathy is a disease of the peripheral nervous system characterized by progressive loss of sensation to pain, temperature, and touch, as well as motor dysfunction. These syndromes can be classified into several processes, including mononeuropathies, multifocal neuropathies, and polyradiculopathies, and may present acutely or over months to years [1].

Radiation-induced brachial plexopathy (RIBP) is one subtype of peripheral neuropathy. It is a known complication of radiotherapy in the head or neck region used to treat several malignancies, including Hodgkin’s lymphoma (HL), lung cancers in the superior sulcus, head and neck cancers, breast cancers, and other neoplasms. Although the incidence of RIBP is 1.2% in patients receiving chemotherapy, it continues to cause significant pain, disability, and morbidity. Its incidence is lowest at cumulative doses less than 40 gray (Gy) but increases markedly as they surpass 45 Gy [2].

Another subtype, chemotherapy-induced peripheral neuropathy (CIPN), is a result of the neurotoxic effects of chemotherapeutic agents. Despite its low incidence in the general population, as more agents are FDA-approved, RIBP is expected to affect an increasing number of people annually [3]. The incidence of CIPN in patients receiving chemotherapy has been estimated to be as high as 68.1% within the first month of the end of treatment, and approximately one-third can expect to develop chronic CIPN at least six months after treatment completion [4]. There are currently no approved treatments for CIPN outside of duloxetine and early chemotherapeutic drug cessation, which risks neoplastic recurrence [5].

CIPN is a well-known complication of brentuximab vedotin (BV), a monoclonal antibody that targets the CD30 surface protein on Reed-Sternberg cells in HL. This agent was approved in November 2022 for the treatment of HL in pediatric patients in combination with doxorubicin, vincristine, etoposide, prednisone, and cyclophosphamide [6].

The double crush phenomenon refers to two sites of nerve compression along a peripheral nerve, resulting in greater neurological dysfunction than at a single site [7]. One example is when CIPN is concomitant with RIBP, although these cases are not well described in the literature. Here, we present a case of RIBP with concomitant BV-induced CIPN who presented to a private academic upper extremity clinic. This case contributes to the literature by describing the diagnosis and clinical characteristics of chemoradiotherapy-related neuropathies and their subsequent management.

## Case presentation

A 16-year-old male, with a diagnosis of refractory chronic HL, presented to our clinic with a four-week history of right upper extremity (RUE) weakness involving the shoulder girdle and elbow flexion. Prior to symptom onset, he was an active member of his high school baseball team.

His initial diagnosis for HL (nodular sclerosis, stage IIA) was two years prior to his current presentation, when he was seen at an outside center for a lump in his right axilla. Four months later, he underwent surgical lymph node removal in his right neck along with four cycles of ABVE-PC (doxorubicin hydrochloride, bleomycin, vincristine sulfate, etoposide phosphate, prednisone, and cyclophosphamide) chemotherapy with an inadequate response. This prompted a total dose of 21 Gy of proton stereotactic body radiotherapy (SBRT) to the neck and mediastinum, achieving complete remission. His first relapse to the neck occurred four months later, which required three cycles of brentuximab/bendamustine. After another three months, a second recurrence confirmed refractory classical HL. He underwent autologous hematopoietic stem cell transplant (HSCT) following BEAM (carmustine, etoposide, cytarabine, melphalan) conditioning. Two months later, he completed 14 treatments of SBRT to the neck with a cumulative dose of 21 Gy; maintenance BV was initiated within another two months. Four months into his maintenance therapy, the patient became significantly sick with influenza A for two to three weeks, as confirmed by a respiratory viral panel. A complete blood count and comprehensive metabolic panel demonstrated no evidence of chronic immunosuppressant use.

**Figure 1:**
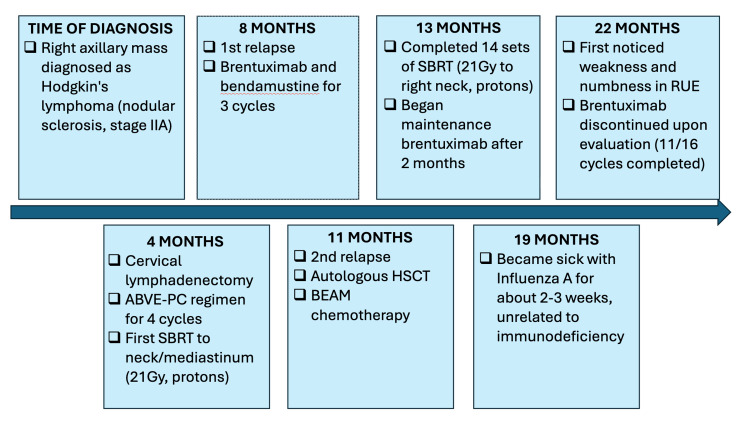
Management of refractory HL ABVE-PC: doxorubicin hydrochloride, bleomycin, vincristine sulfate, etoposide phosphate, prednisone, and cyclophosphamide; HSCT: hematopoietic stem cell transplant; BEAM: armustine, etoposide, cytarabine, melphalan; SBRT: stereotactic body radiotherapy; RUE: right upper extremity, HL: Hodgkin’s lymphoma

Although he had previously received several potentially neurotoxic radiotherapeutic and chemotherapeutic agents, at the time of BV initiation, he had no symptoms to suggest the presence of peripheral neuropathy. However, nine months after his last course of radiation, the patient began experiencing right-hand numbness and weakness with elbow and finger flexion. Maintenance brentuximab was discontinued, resulting in an incomplete treatment cycle. Figure 1 summarizes the patient's clinical course for HL.

Manual muscle testing using the Medical Research Council scale was performed to assess bilateral upper-extremity strength. The patient exhibited 4/5 right elbow and finger flexion; he was able to flex his elbow and fingers against gravity and limited resistance, but not against maximum resistance. Semmes-Weinstein monofilament testing was performed to assess light touch sensation and sensory nerve function. The right thumb and middle finger demonstrated decreased sensation, requiring 3.61 monofilament compared to normal sensation (2.83 monofilament) in the remaining digits. Otherwise, his vascular status was normal, with 2+ radial and ulnar pulses and capillary refill of less than two seconds. An MRI of the cervical spine and brachial plexus was ordered with and without contrast to rule out neoplasia. The patient was tentatively diagnosed with right upper arm neuritis and advised to consult with his oncologist and a neurologist to further investigate his symptoms/correlate these with his extensive HL history.

Investigations and treatment

**Figure 2:**
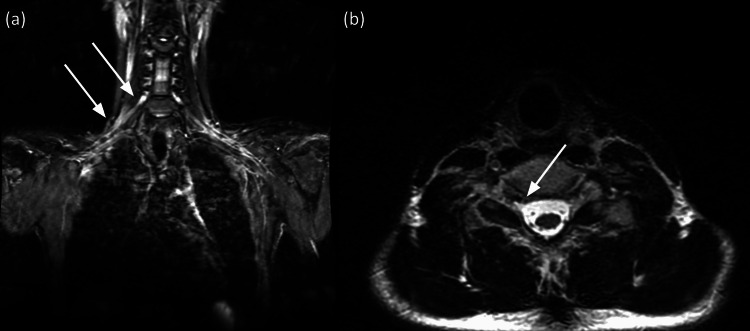
MRI of the brachial plexus and cervical spine (A) Coronal T2 STIR MRI of the brachial plexus. (B) Axial CUBE T2 MRI of the cervical spine. STIR: short tau inversion recovery; CUBE: coherent, ultrafast, beam, echoes; MRI: magnetic resonance imaging

The patient’s MRI was performed within a week of this initial visit. MRI of the cervical spine and brachial plexus with or without contrast demonstrated asymmetric thickening of the right C5-C8 nerve roots and trunks with a high T2 signal and subtle enhancement with contrast (Figure 2). Otherwise, no discrete or intrusive mass was identified within the brachial plexus, and these findings were correlated with chronic inflammatory demyelinating polyradiculopathy (CIDP).

A month later, the patient returned to the clinic with progressive weakness and atrophy of more muscle groups in the RUE and chest wall. He described no change in numbness and no new pain. On this visit, we reviewed prior electromyography and nerve conduction studies (EMG/NCS) evaluating peripheral nerve function. These were performed at a secondary institution and revealed nerve abnormalities in his lower extremities, although he reported no foot symptoms. In the interim, he had discontinued his brentuximab cycles and initiated physical therapy and exercises at home.

The decision was made to continue nonoperative management, and the patient was prescribed a course of PO prednisone 30 mg BID × 30 days. We had considered using IVIG and plasmapheresis in case of treatment failure, as well as a biopsy to rule out neoplastic syndrome. It was explained to the patient that if he were to have significant irreversible motor impairment or intolerable neuropathic pain, surgical management may be offered as a last resort in the form of a neurolysis or a hemi-latissimus, free omental, or temporoparietal fascial flap. In the meantime, the patient would obtain an updated EMG/NCS and follow up in three months or sooner if symptoms progressed.

This updated EMG/NCS demonstrated generalized primary motor and borderline-to-low-mild sensory primary axonopathic processes, with low-mild demyelinating changes involving the right upper limb more than the left and the left lower limb. These findings indicated a generalized right-sided plexopathic process, although no myokymic potentials were identified. Compared to prior EMG/NCS, there were no signs of active denervation in the affected distributions. By contrast, evidence of reinnervation was observed in the affected muscle groups. Moreover, the changes observed on the left correlated with the patient's reported improvement in left-extremity weakness at follow-up. Given evidence of reinnervation and clinical improvement, the patient was advised to continue nonoperative management of his symptoms and to follow up as needed. Overall, these electroclinical findings suggested a motor neuronopathy, a polyradiculoneuropathic process, or a component of a polyradiculoneuropathic process.

**Figure 3:**
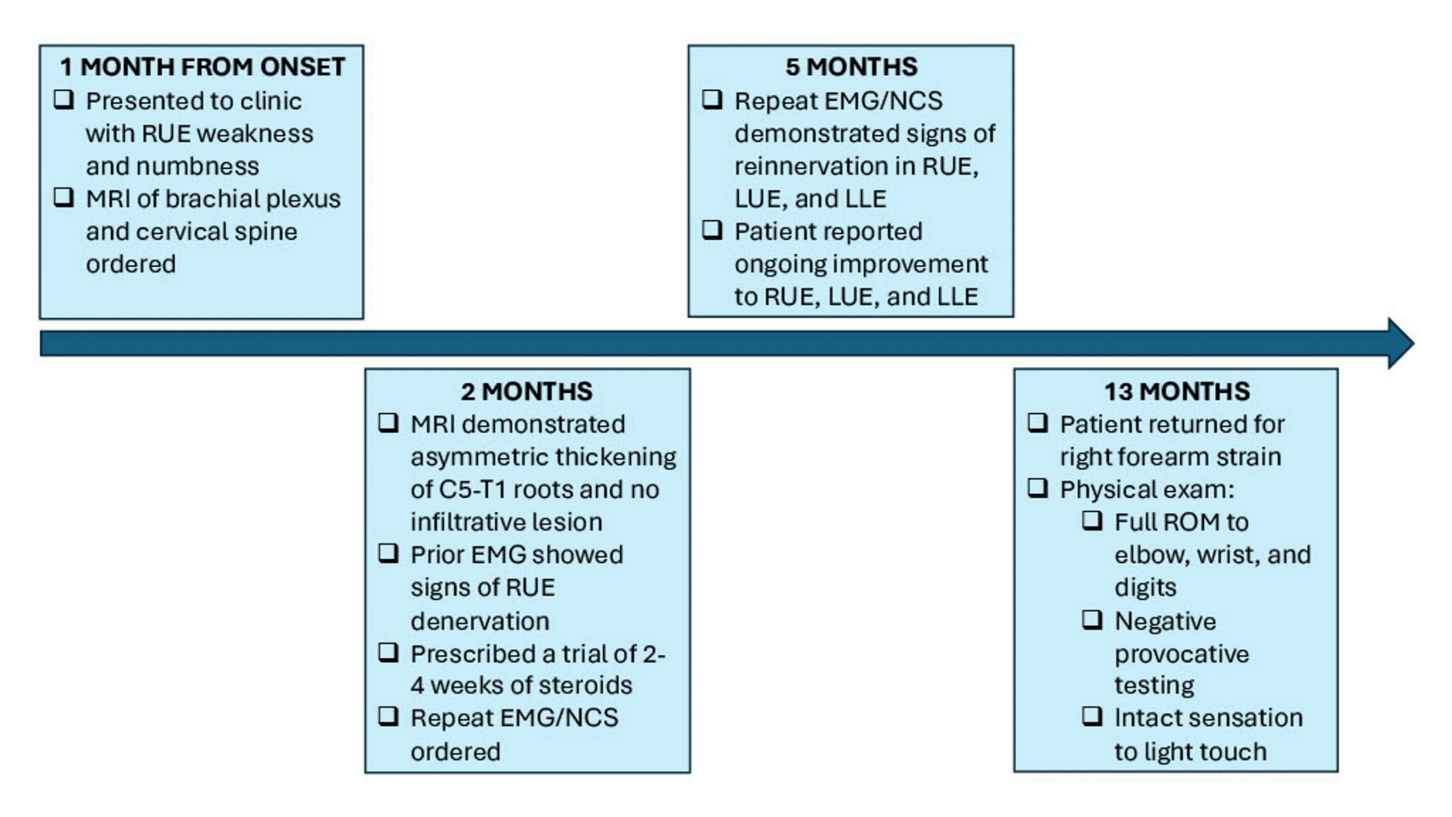
Management of RUE weakness RUE: right upper extremity, LUE: left upper extremity, LLE: left lower extremity, EMG/NCS: electromyography and nerve conduction studies

Eight months after reviewing his updated EMG/NCS and 13 months following his initial complaint, the patient followed up with our center to discuss management of a right forearm contusion/proximal forearm strain. His current symptoms and physical examination demonstrated no weakness on resistance and intact sensation to light touch, suggesting that he had otherwise fully recovered. Figure 3 summarizes the interventions at our center.

Differential diagnosis

Our patient initially presented with a non-length-dependent, painless, primarily motor neuropathy beginning in his RUE. We suspected that this was RIBP or Parsonage-Turner syndrome, given his history of stereotactic radiation therapy to the right side of his neck. However, his presentation was unusual, as he had complained of motor-predominant symptoms without significant pain or numbness, which would be seen in Parsonage-Turner syndrome [8].

Moreover, given that the patient began to experience symptoms on his left side and in his lower extremities, his symptoms were thought to be unrelated to his radiation therapy, which was isolated to his right side. Additionally, he lacked myokymic potentials, which are typical of lower motor neuron lesions, such as RIBP [2,9]. Thus, his symptoms could be related to an inflammatory phenomenon, potentially paraneoplastic from his refractory HL, or chemotherapy-related to BV, which has been implicated as a neurotoxic agent. Given our suspicion of a paraneoplastic syndrome, we consulted with his oncologist to consider a biopsy in the future, even though his MRI images did not demonstrate any compressive masses.

We also considered the contribution that the patient’s influenza A infection may have made to his presentation. Several reports, although underpowered, demonstrate that both influenza infection and influenza vaccination are associated with Guillain-Barré Syndrome (GBS), a symmetric, length-dependent, demyelinating polyradiculoneuropathy characterized by ascending paralysis [10]. Of note, the patient experienced primarily motor symptoms with sensory sparing; he also demonstrated EMG/NCS changes that were predominantly axonopathic. Together, this could represent a subtype of GBS known as acute motor axonal neuropathy (AMAN).

However, GBS was unlikely, as the patient’s symptoms initially presented in his RUE, and reports often cite the risk of respiratory paralysis in cases of AMAN [11]. Moreover, corticosteroids have not been described to have a beneficial effect on GBS and its variants [12]. This patient had resolution of his symptoms with a course of prednisone and physical therapy.

This patient demonstrated radicular symptoms in the brachial plexus with cervical nerve root thickening and enhancement per MRI. However, he also showed demyelinating changes across multiple extremities suggestive of a peripheral neuropathy, which has been associated with brentuximab [13]. We propose that this is a case of RIBP complicated by brentuximab-induced peripheral neuropathy, wherein radiation to the brachial plexus allowed the neurotoxic effects of brentuximab to preferentially affect the RUE, hence the initial presenting symptoms.

## Discussion

Our patient presented with a chronic-onset, non-length-dependent, painless motor neuropathy, beginning in his RUE eight months following treatment with BV and subsequently involving the left side and his lower extremities. Nerve conduction studies were consistent with CIDP and demonstrated significant axonal loss. There was no evidence to support neoplastic infiltration according to his MRI of the brachial plexus and cervical spine.

Our patient presented with primarily motor rather than sensory symptoms affecting his RUE before involving his other limbs. Radiotherapy to the neck and axilla can cause RIBP, which typically presents with predominantly motor dysfunction of the upper limb rather than sensory loss, as seen with CIPN [14]. However, CIPN is a compelling consideration given the patient's involvement of multiple extremities.

The pathophysiology of RIBP has been hypothesized to involve damage to the brachial plexus vasculature, leading to ischemia and subsequent nerve injury. Radiation can also directly damage nerve tissue, triggering a cascade of inflammation and fibrosis that impairs sensory and motor functions [9].

In addition to the cascade initiated by radiotherapy, our patient’s symptoms were likely compounded by axonopathy produced by the neurotoxic effects of brentuximab. The mechanism for these effects has not been fully elucidated. Pastorelli et al. suggest that brentuximab exerts neurotoxic effects by inhibiting microtubule-dependent axonal transport, much like vinca alkaloids [15]. Other studies suggest that the anti-CD30 antibody may induce an indiscriminate autoimmune response targeting all CD30-expressing cells, which may explain the positive outcomes observed with IVIG and plasmapheresis [13,15,16]. Brentuximab has been previously described to cause peripheral neuropathy in 60-70% of patients, although most symptoms have been sensory rather than motor [13]. Flerlage et al. conducted a pharmacokinetic study to develop risk profiles for pediatric patients at risk of neuropathy as a complication. They found that weight and sex may alter clearance rates, thereby increasing the risk of neuropathy [17]. Nonetheless, despite its incidence, this is a safe chemotherapeutic agent according to reports on three-year progression-free survival and overall survival, both as an additive agent and as monotherapy [18]. Physicians should adjust their index of suspicion based on patient risk factors, given that the risk-to-benefit ratio of brentuximab in HL is low.

The double crush phenomenon accounts for our patient’s presentation: neuritis of the brachial plexus following radiation therapy represented the first “hit,” and brentuximab produced the second “hit,” resulting in the initial presentation. Chemotherapeutic agents may have revealed or amplified radiation-related nerve injury that was previously asymptomatic [7]. Clinicians should maintain a high index of suspicion for the cumulative stress that multiple instances of local radiotherapy and chemotherapy can have on the peripheral nervous system.

There is little literature on isolated therapies that may specifically prevent BV-induced neurotoxicity. Instead, initial therapy is likely to be supportive and intended to treat neuropathic pain. Given its immunosuppressive effects and its modulation of inflammation within the fibrotic cascade, prednisone may be an appropriate therapy for the management of peripheral neuropathy associated with chemotherapy/monoclonal antibody treatment, as evidenced by the relief our patient experienced [19,20]. Large prospective studies are necessary to determine its efficacy in this setting.

This study lacks precise quantitative dosing data, which limits the generalizability of these findings, and any interpretations should be treated as hypothesis-generating. Several more comprehensive studies have identified risk factors for RIBP and the optimal radiation dose and schedule. Unfortunately, few studies examine the precise intersection of effects on patients who underwent both radiotherapy and chemotherapy. Future studies may systematically analyze radiation doses in combination with various chemotherapy regimens. However, such analyses may be limited by small sample sizes and limited data, given the marked decrease in RIBP incidence.

This case report further adds to the current evidence, as it is important to better characterize the clinical characteristics of cancer-related neuropathies to understand the neurotoxic effects of therapies.

## Conclusions

As chemoradiotherapy protocols continue to be refined and morbidity from cancer decreases, the number of patients presenting with the double crush phenomenon is likely to increase. Neurotoxic chemotherapeutic agents potentiate the effects of localized radiotherapy, initially producing a localized deficit that subsequently extends peripherally. Clinicians should maintain a high index of suspicion in patients receiving combined chemotherapy and localized radiotherapy. Serial nerve conduction studies may help determine whether a deficit is truly isolated or represents the early stages of a generalized process. Further studies are required to determine which patients receiving combined therapy are at risk of the double crush phenomenon and whether dose reduction is safe and warranted to improve long-term functional outcomes.
